# Dentist’s Delay or Dexterity to Diagnose the Deadly: A Clinico-radiological Series of Oral Malignancies Exhibiting Varied Presentations in the Tamil Nadu Rural Belt

**DOI:** 10.7759/cureus.4051

**Published:** 2019-02-11

**Authors:** Praveena Raman, Ponnusamy Subramani Gayathri

**Affiliations:** 1 Oral Medicine and Radiology, Sathyabama Dental College and Hospital, Chennai, IND; 2 Oral Medicine and Radiology, Thai Moogambigai Dental College and Hospital, Chennai, IND

**Keywords:** oral cancer, squamous cell carcinoma, malignancy, delay diagnosis, contrast enhanced computed tomography, ulcero-proliferative growth, early diagnosis, public awareness, health care professionals, tobacco

## Abstract

Oral cancer is the sixth most common malignancy globally with a wide geographic variation. India is the second largest consumer and third largest producer of tobacco in the world. One-third of the global burden of oral cancer is predominantly attributed to high prevalence of tobacco consumption. The highest incidence and prevalence of oral squamous cell carcinoma is found in the Indian subcontinent particularly in the lower socioeconomic strata, due to an increase in the deleterious habits of potent, proven carcinogens like smoking, chewing tobacco, betel quid and areca-nut. Also, there is a delayed presentation of oral cancer in India, as approximately 50% of patients are diagnosed at stage III or IV. In this article, we report five varied presentations of well differentiated oral squamous cell carcinoma from rural belt of Tamil Nadu. All the cases were reported late to diagnose. Clinical and radiological staging plays a pivotal role to stage an oral malignant patient which aids in guiding him to a proper treatment plan. Early diagnosis along with patient counselling is of vital importance for the prognosis of the patients with oral malignancies. Also, it is an utmost important duty of the health care professionals to create awareness on oral cancer especially in rural areas. For this reason, dentists play a very significant role in the early detection and prevention of oral malignancies.

## Introduction

Oral cancer is the sixth most common malignancy worldwide [1]. Ninety percent of oral malignancies constitutes oral squamous cell carcinoma (OSCC) [2]. India has one of the highest incidences of oral cancer and about 30% of new cases are reported annually [3]. The highly proposed risk factors for oral cancer include habits of tobacco; areca nut; alcohol, immune-compromised state, dietary habits, and infection with certain types of human papillomaviruses (HPV) [4]. It is pertinent to mention that, most of the cases reported are late to diagnose, which can either be from the patient side or a professional side. The treatment options for OSCCs include a combination of surgery, chemotherapy and radiotherapy depending on the final histopathological results [5].

Dentists play a significant role in the early detection, counselling and prevention of oral malignancies. This article presents a detailed description of the varied presentations of five patients diagnosed with well differentiated OSCC in rural belt of Tamil Nadu. All patients belong to lower socio-economic rural background and all of them were late to diagnose.

## Case presentation

Case 1

**Figure 1:**
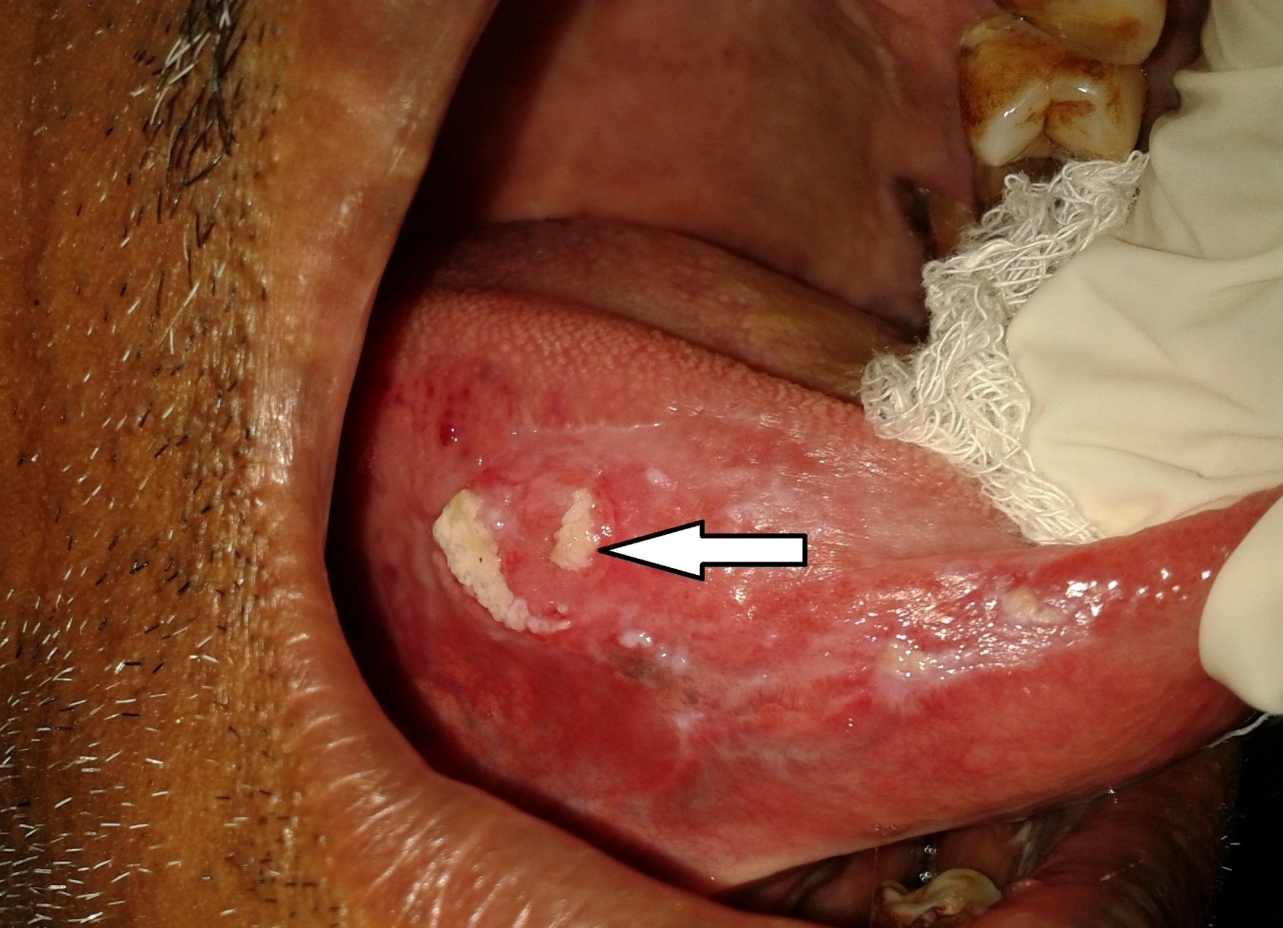
Clinical image of proliferative verrucous leukoplakia transforming to a squamous cell carcinoma involving right lateral border of tongue.

**Figure 2:**
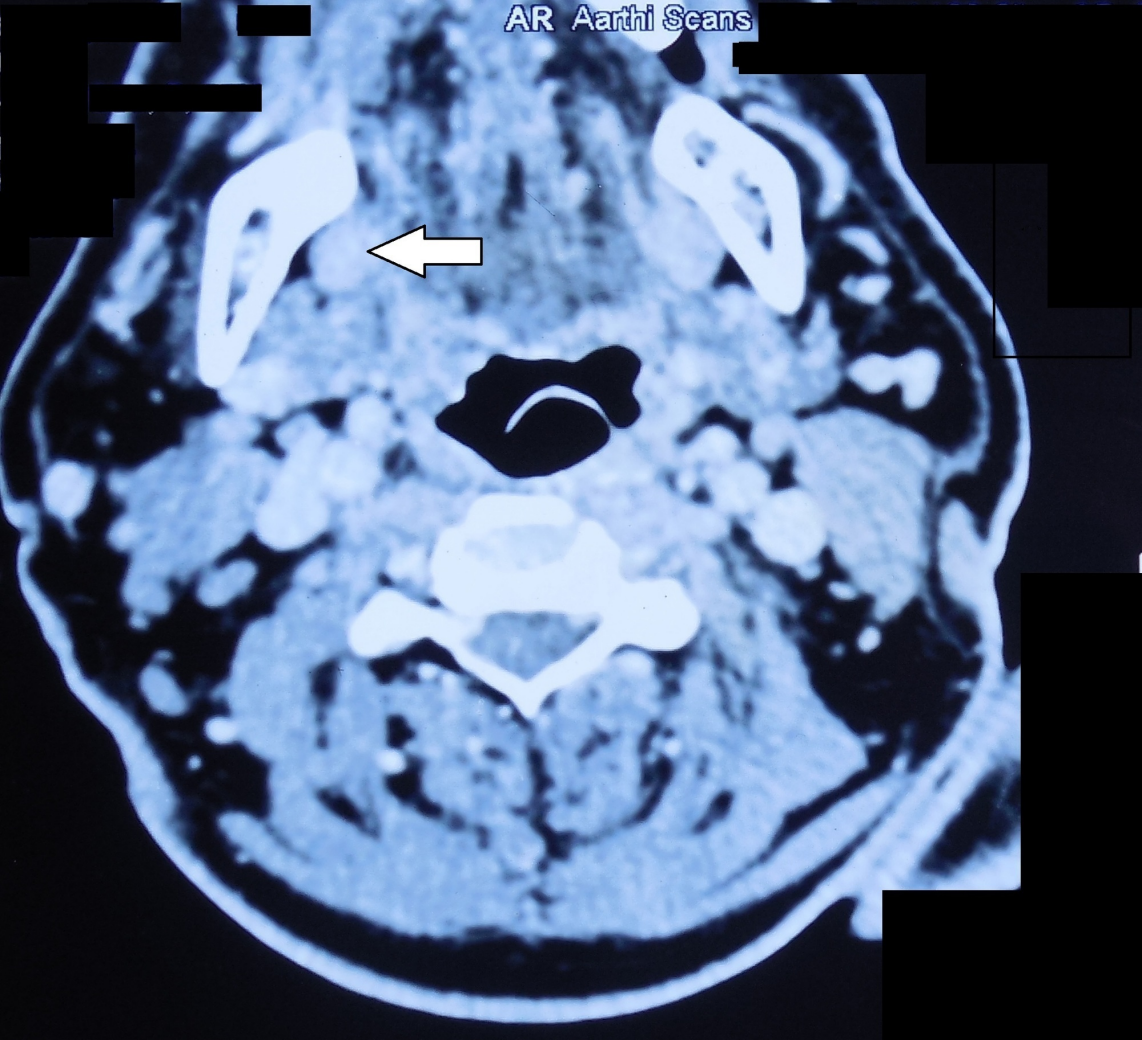
Contrast-enhanced computed tomography axial section showing mild heterogenous contrast enhancement in the right lateral border of tongue and few prominent lymph nodes at level I and II bilaterally with preserved fatty hilum.

A 55-year-old male patient complained of burning sensation at right side tongue region for one month. The patient had a history of betel nut chewing for 30 years (10 betel nuts/day). The patient’s father passed away due to oral cancer 15 years back. Intraoral examination revealed a tender, firm, 2 × 2.5 cm ulcero-proliferative growth at right lateral border of tongue with normal tongue movements as shown in Figure 1. A single 1 × 1 cm tender, firm, ovoid lymph node was palpable at the right submandibular region. A provisional diagnosis of proliferative verrucous leukoplakia was made. Figure 2 illustrates the contrast-enhanced computed tomography (CECT) findings. Biopsy confirmed infiltrating squamous cell carcinoma (SCC) involving right lateral border of tongue. Tumour node metastasis (TNM) staging: III- T2 N1 M0.

Case 2

**Figure 3:**
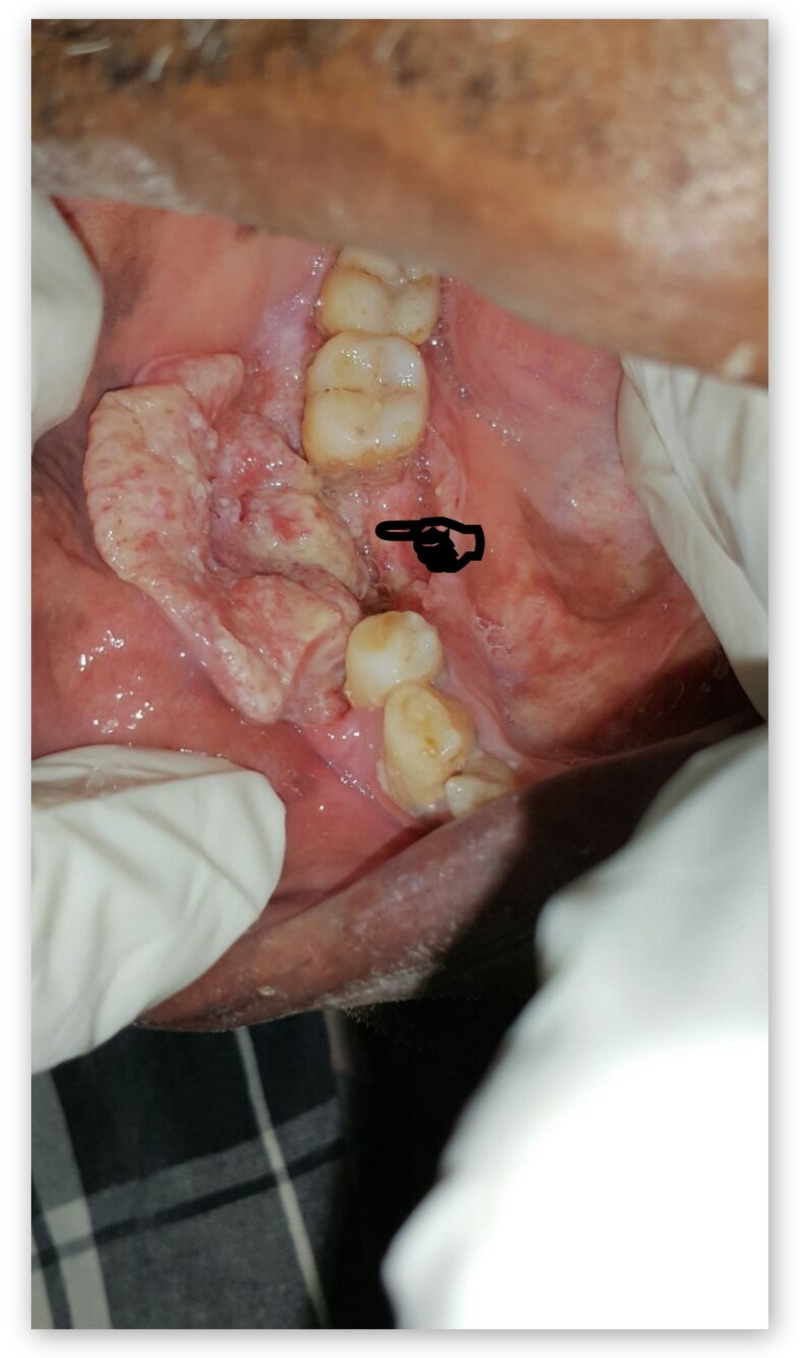
Clinical image of the malignant ulcer involving right alveolus and buccal region.

**Figure 4:**
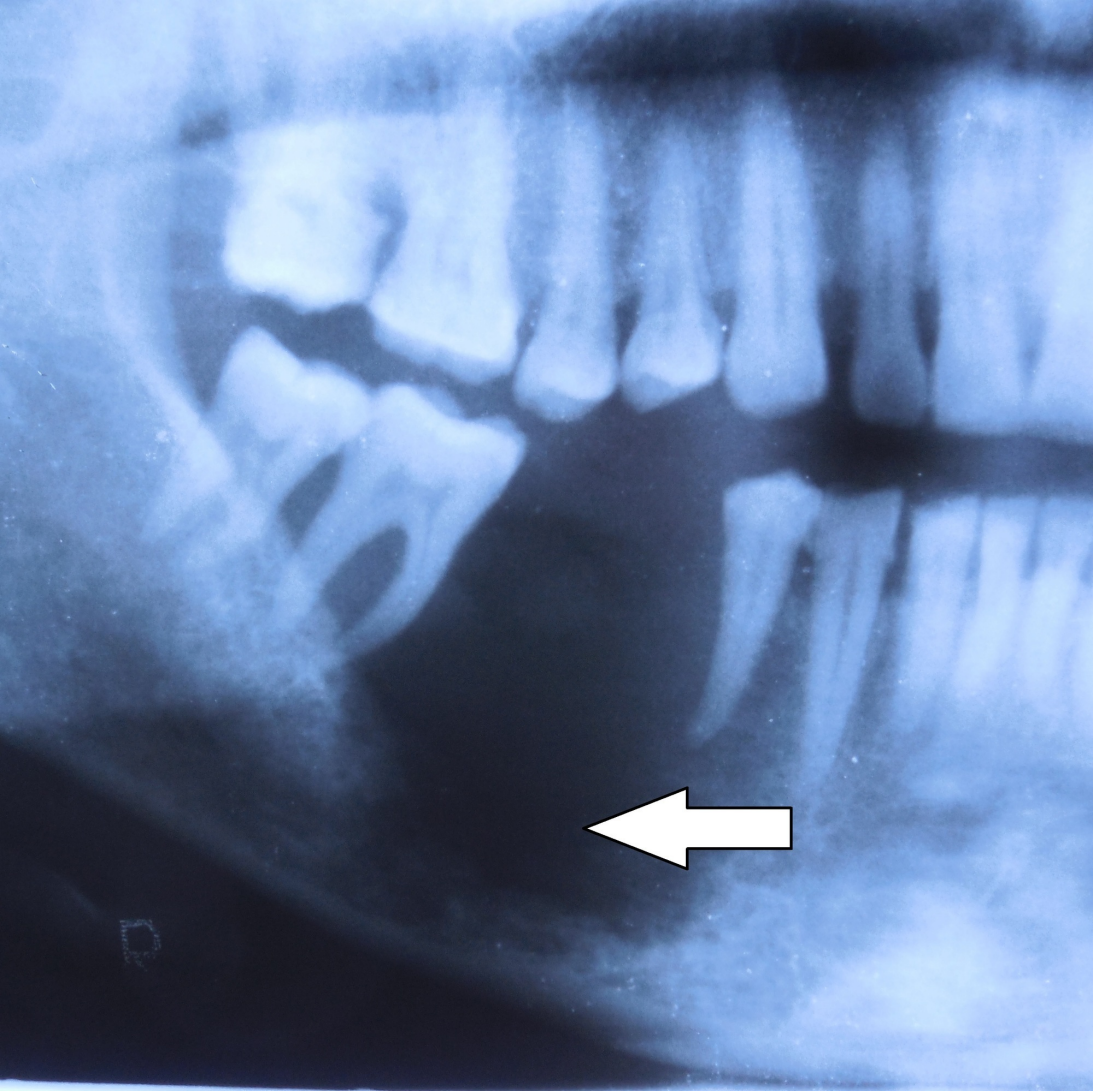
Cropped panoramic view showing ill-defined invasive borders at right posterior alveolar bone in relation to the edentulous region at 45,46 with erosion of the underlying bone. A totally radiolucent internal structure with no evidence of normal trabecular bone. Teeth 45 and 47 appear to float in a mass of radiolucent soft tissue bereft of any bone support with thinning of the inferior border of the mandible.

**Figure 5:**
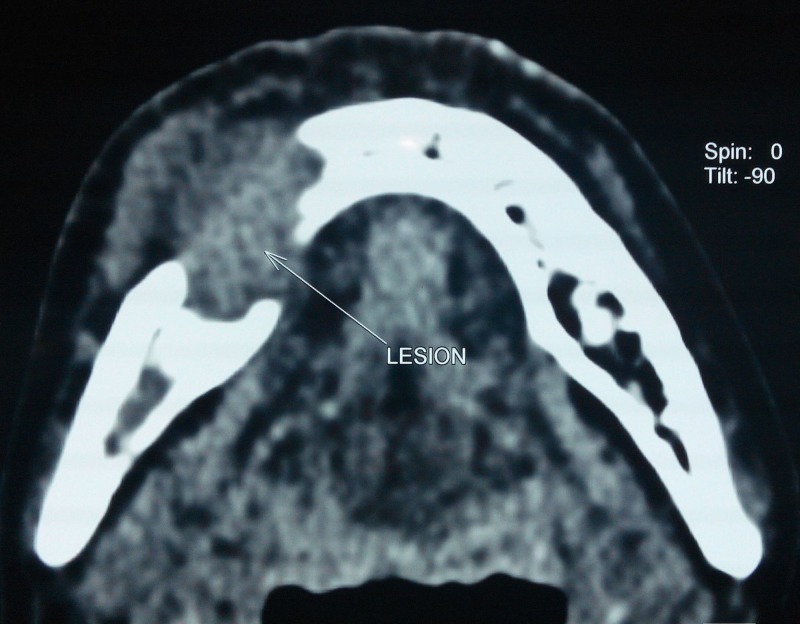
Axial section of plain computed tomographic image showing a 4.4 × 3.7 × 2.6 cm size ill-defined well-enhancing soft tissue density lesion at anterior aspect of body of mandible, gingival mucosa, right lower gingiva-buccal sulcus and buccal mucosa, causing lysis of mandible with erosion involving both buccal and lingual cortex with loss of fat plane between the lesion and right mylohyoid muscle.

**Figure 6:**
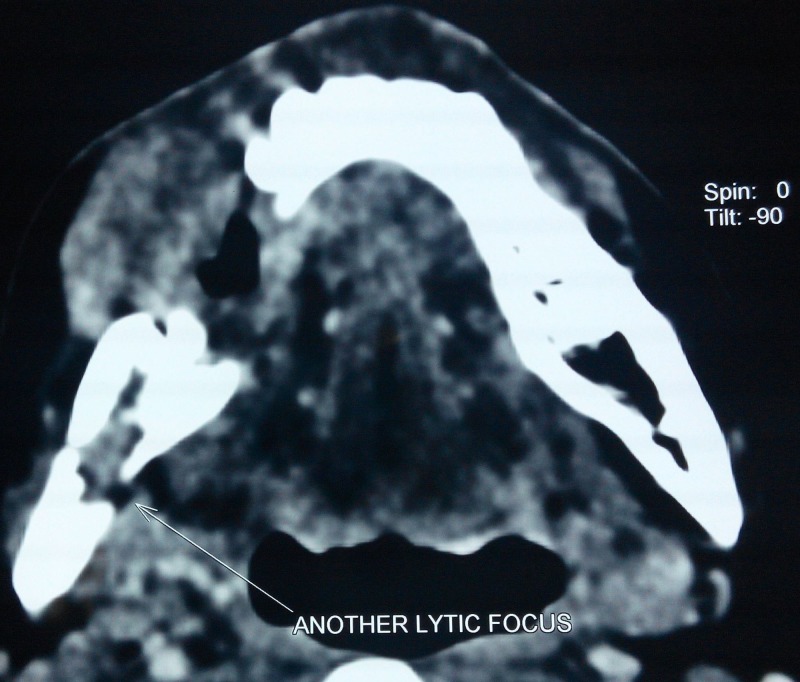
Axial section of computed tomographic image showing another 1 × 1.7 cm ill-defined well-enhancing lytic lesion at right angle of mandible with erosion of lingual cortex.

**Figure 7:**
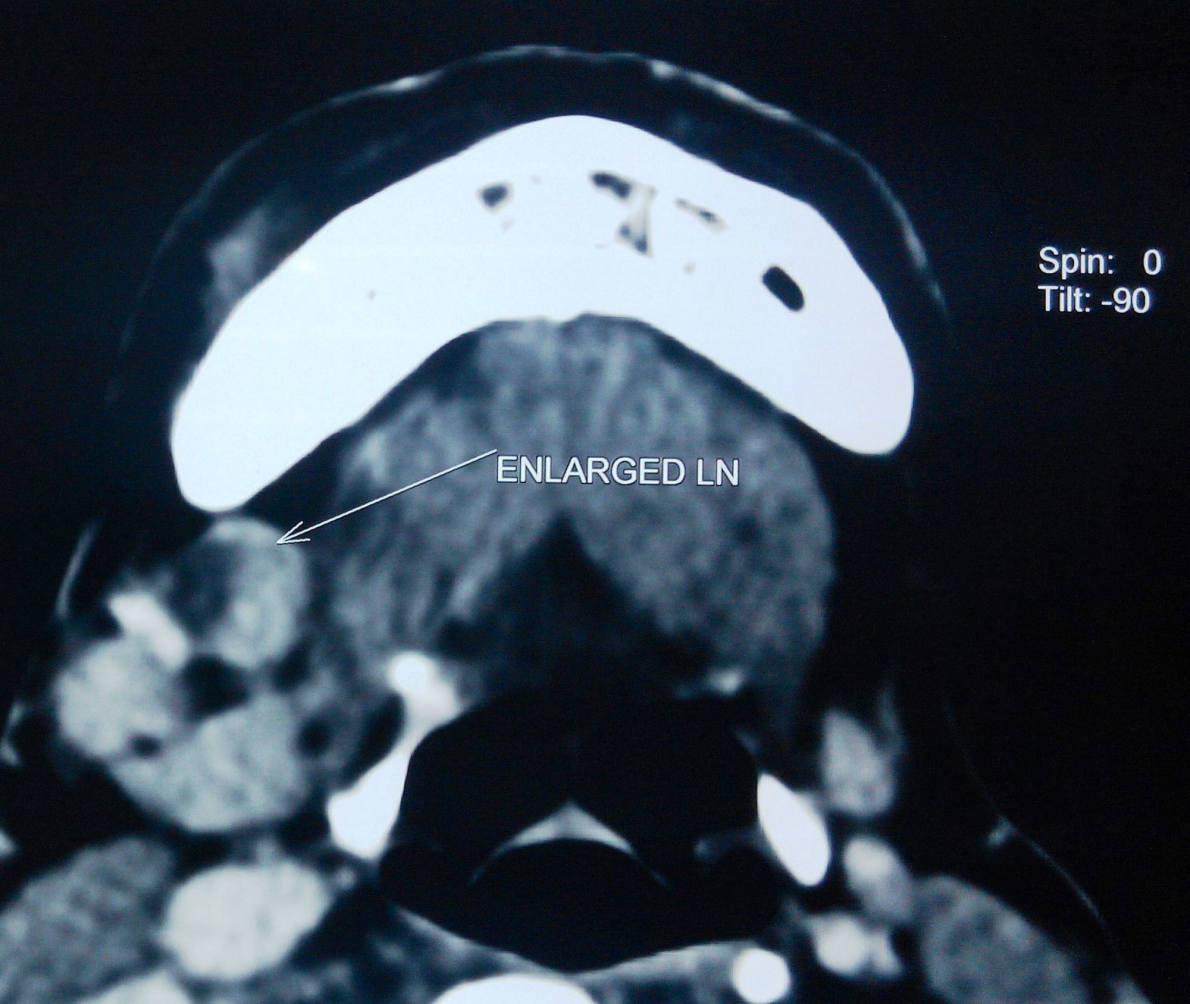
Axial section of contrast-enhanced computed tomographic image showing few prominent lymph nodes at level IA and IB of right side of neck (largest measuring 1.7 × 1.3 cm), one of the prominent lymph nodes showing necrosis within it.

A 65-year-old male patient complained of painful growth at right lower back tooth region for 15 days. The patient had a history of chewing pan for the past 15 years (3-5 packets/day). Intraoral examination revealed a 4 × 3 cm, tender, hard ulceroproliferative growth, arising from the edentulous ridge of 45,46 extending into the alveolus and right buccal mucosa as shown in Figure 3. Rolled out edge and an indurated base were confirmed on palpation. A single 3 cm × 2 cm, well-defined hemispherical-shaped tender, firm, fixed lymph node was palpable at the right submandibular region. A provisional diagnosis of malignant ulcer involving right alveolus and buccal region in relation to 45,46 was made. Figure 4 shows the panoramic view. Figures 5-7 show all the CECT findings which were suggestive of malignant mass lesion with lymph node secondaries. Biopsy confirmed well-differentiated SCC. TNM staging: IVA- T4a N1 M0.

Case 3

**Figure 8:**
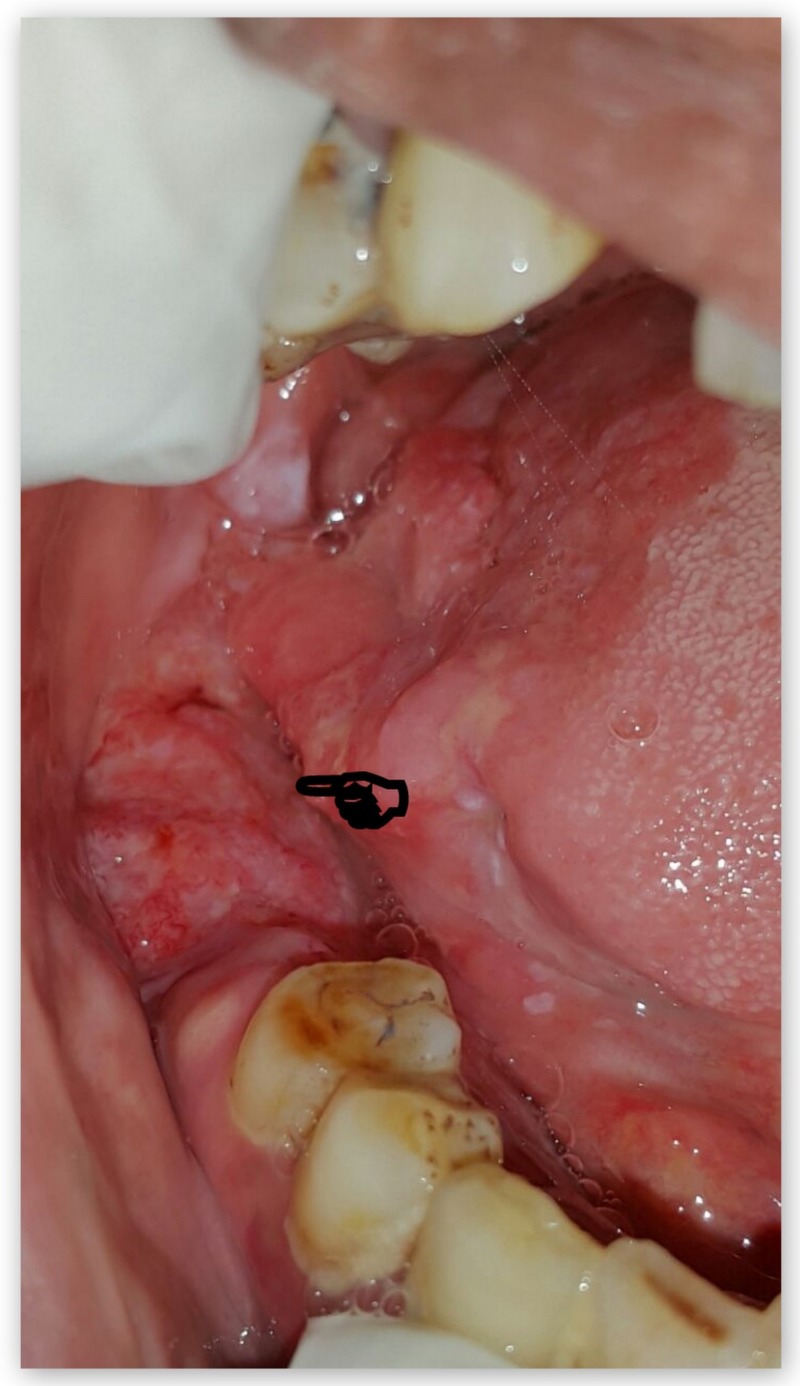
Clinical image showing an ulcero-proliferative growth involving right posterior alveolar ridge and right lateral border of tongue.

**Figure 9:**
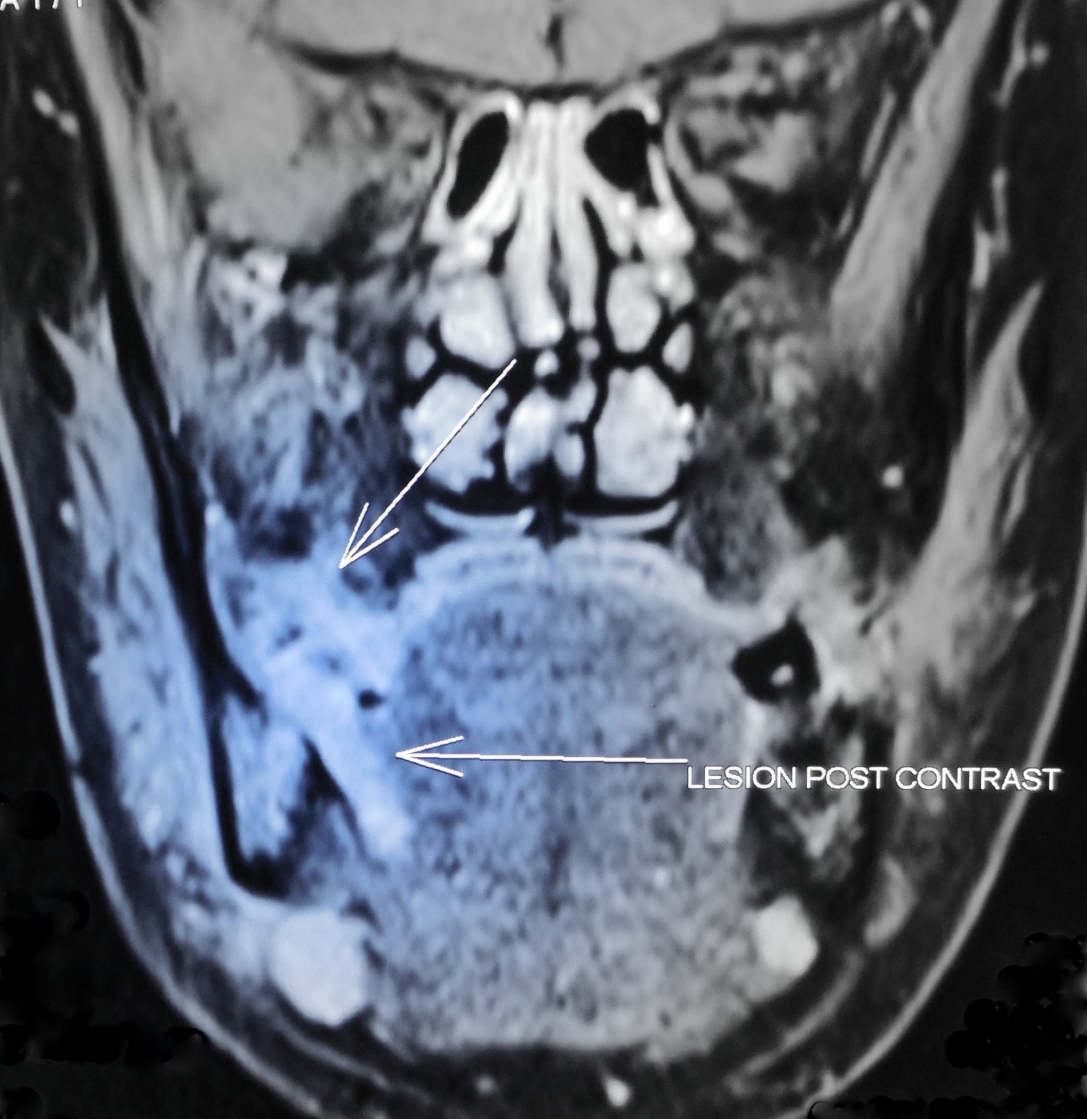
Contrast magnetic resonance imaging, coronal section showing ill-defined, irregular-shaped, well-enhancing hyperintense lesion approximately measuring 2.6 × 1.8 × 1.5 cm at right mandibular alveolar process extending into adjacent gingival mucosa, right lateral aspect of tongue and right side floor of mouth involving right mylohyoid muscle. The lesion causes erosion of posterior aspect of body of mandible. Multiple enlarged lymph nodes were noted in level I of both sides neck (largest measuring 1.6 × 1.3 cm) with no significant evidence of necrosis in enlarged lymph nodes.

**Figure 10:**
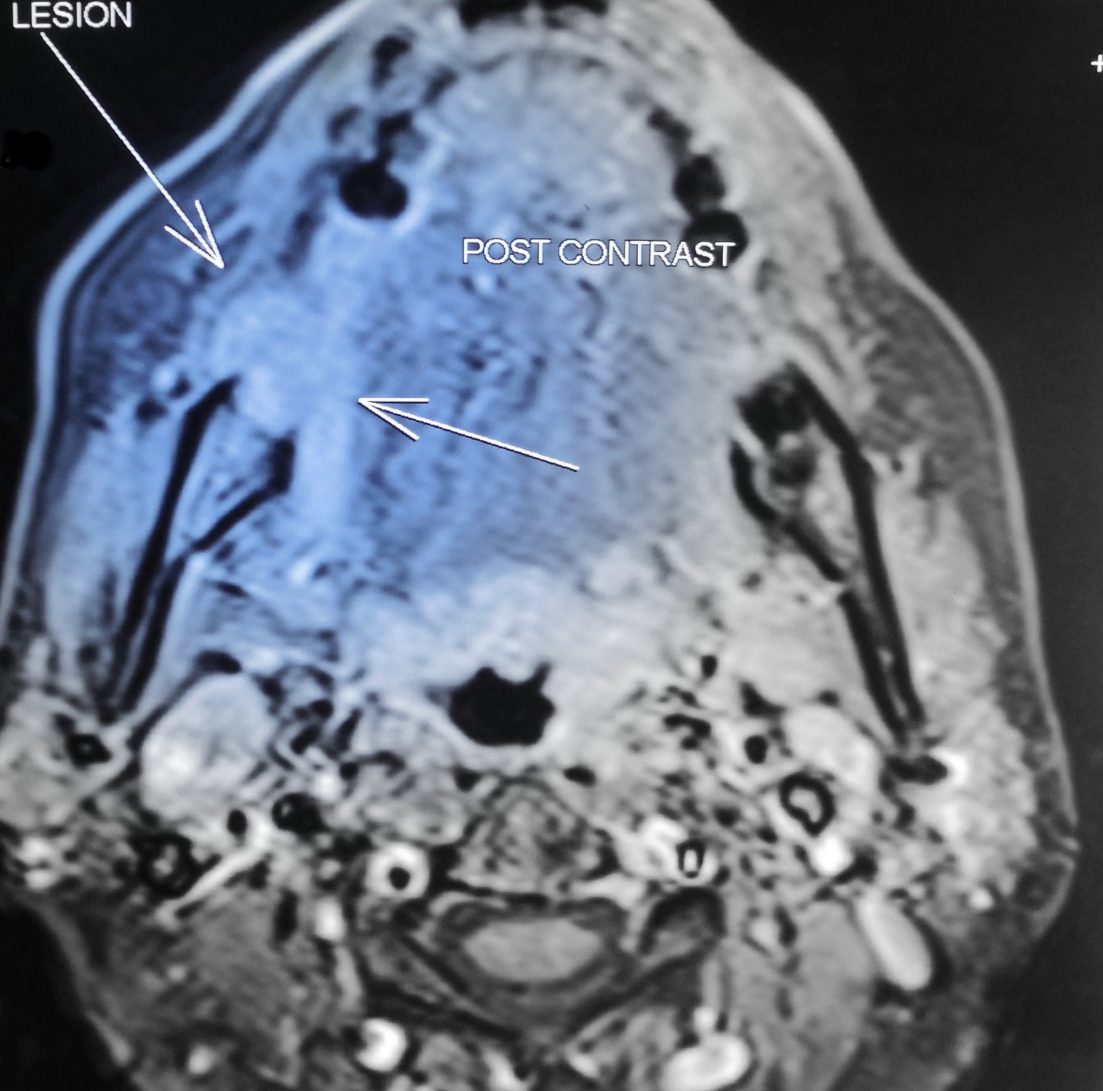
Contrast magnetic resonance image - axial section.

A 65-year-old female patient complained of pain and swelling at right lower back gum and tongue region for 10 days. The patient had a history of similar intensity of pain along with burning sensation at right side tongue region two years back, for which she visited a nearby dental practitioner at her home town in a small village and excisional biopsy was done. The histopathological report had revealed early features of malignancy with severe dysplasia for which patient was prescribed a course of antioxidants and multivitamins for one month due to improper medical facilities. Afterwards, the patient was completely asymptomatic for nearly two years. The patient had no history of usage of any form of tobacco or alcohol. Intraoral inspection revealed a 3 × 3.5 cm hard, ulceroproliferative growth on the edentulous alveolar ridge mucosa of tooth 46,47,48. A single 3 × 3.5 cm hard, indurated, irregularly shaped, lobulated growth evident at the right lateral border of tongue with restricted tongue movements is shown in Figure 8. Tender, hard, fixed, hemispherical-shaped lymph nodes of 1 × 1 cm were palpable at the right submandibular region. A provisional diagnosis of malignant growth was made. Contrast magnetic resonance imaging (MRI) was suggestive of neoplastic lesion with metastatic lymph nodes as shown in Figures 9-10. Biopsy confirmed SCC involving right lateral border of tongue, right retromolar trigone and right posterior alveolar ridge. TNM staging: IVA- T4a N1 M0.

Case 4

**Figure 11:**
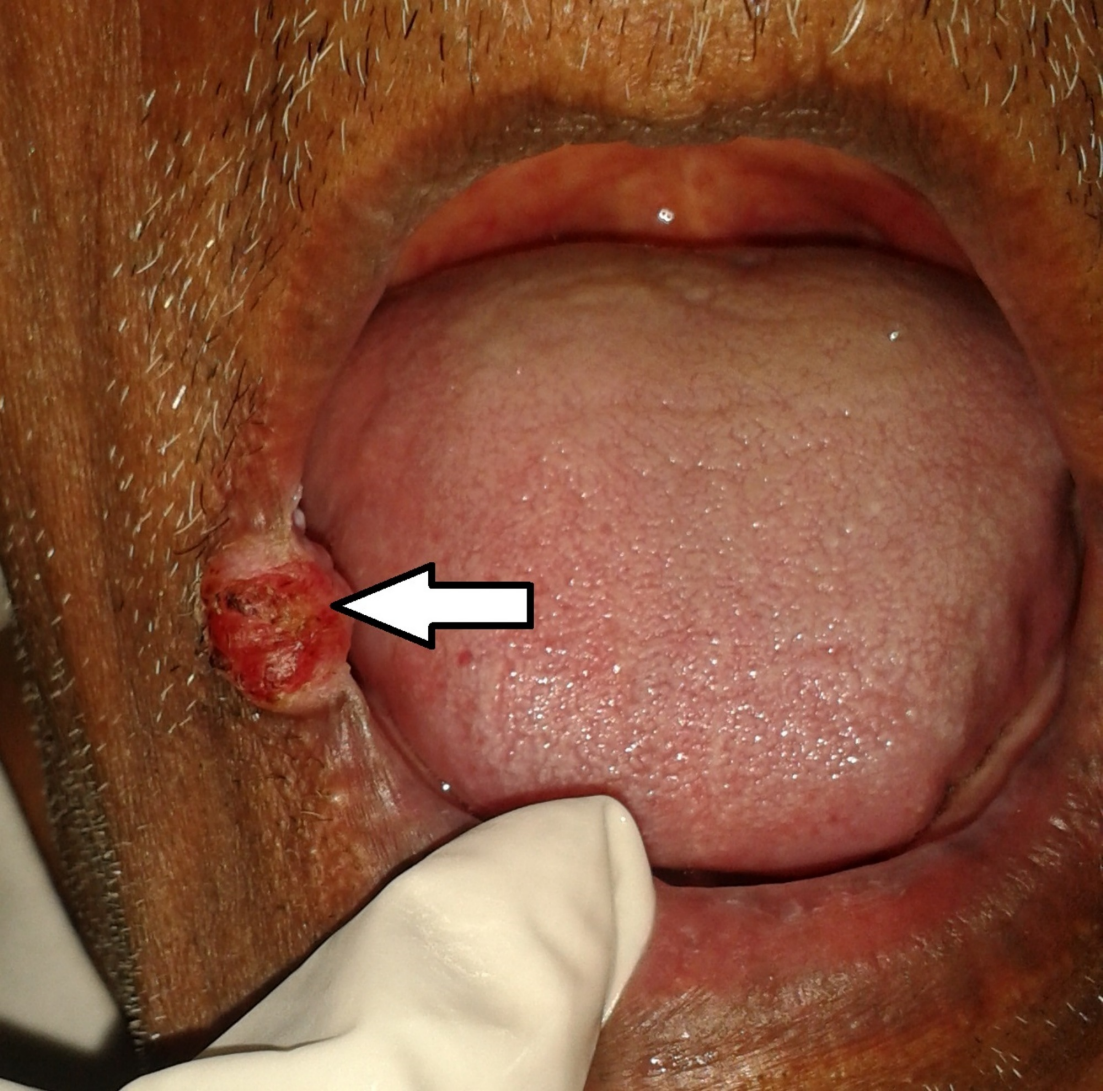
Clinical image of an ulcero-proliferative growth at right commissure.

**Figure 12:**
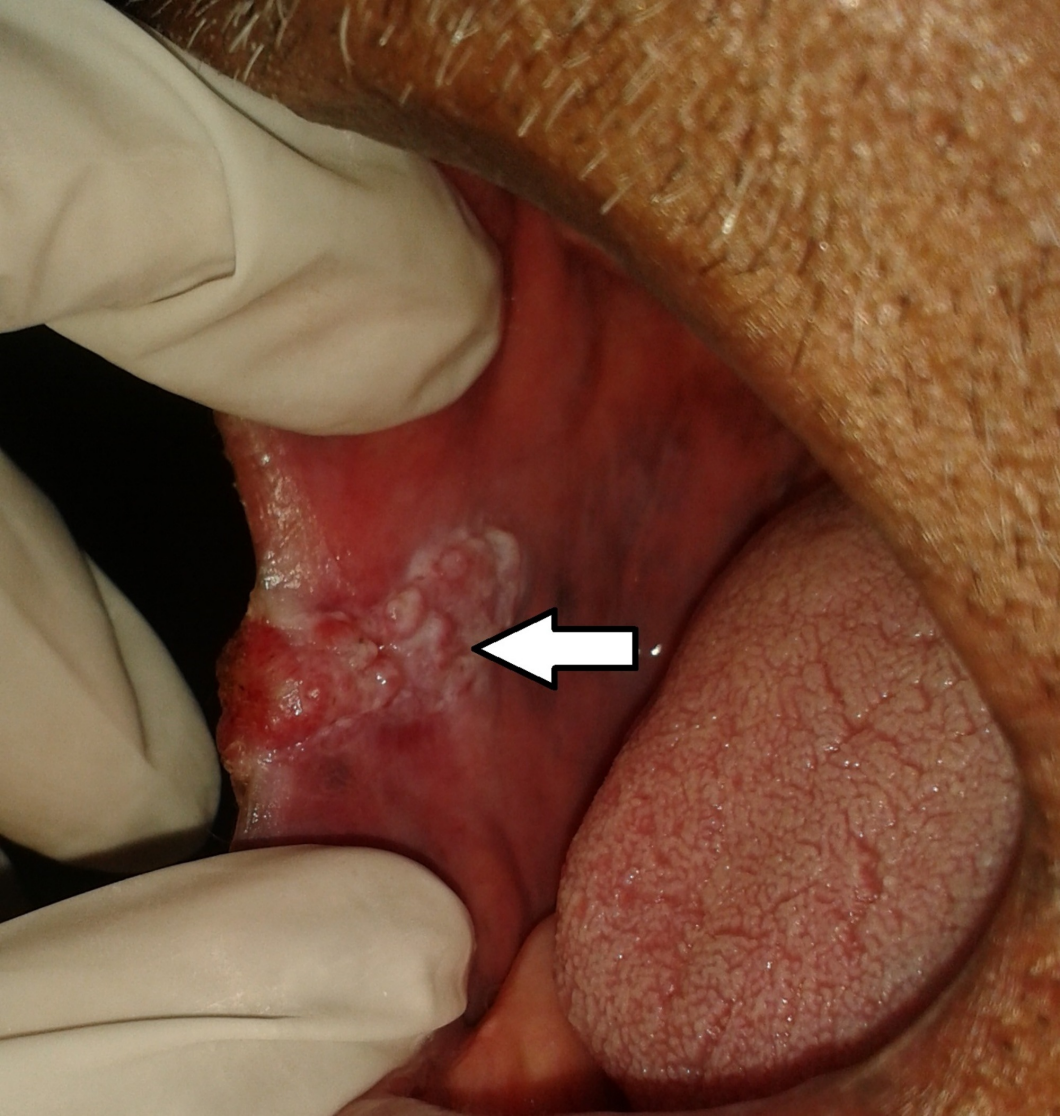
Clinical image of an ulcero-proliferative growth at right commissure extending intraorally.

A 77-year-old male patient complained of a painful non-healing growth at his right side corner of lip for three months. The patient had a history of smoking for 43 years (4-5 cigarettes/day). Extraoral examination revealed a single solitary well-defined, erythematous, tender, hard, indurated oval-shaped ulcero-proliferative growth at the right side lip region at the corner of mouth as shown in Figures 11-12. A single 0.3 x 0.4 cm tender, hard and fixed lymph node was palpable at right submandibular region. Intraoral examination revealed a hard, tender, ulcero-proliferative growth at right commissure region approximately measuring 5 x 1 cm extending into the left buccal mucosa at the level of the occlusal plane. Tender, hard, fixed, hemispherical-shaped lymph nodes of 1 × 1 cm were palpable at the right submandibular region. A provisional diagnosis of malignant, non-healing ulcer was made. Biopsy confirmed well-differentiated SCC. TNM staging: IVA- T4a N1 M0.

Case 5

**Figure 13:**
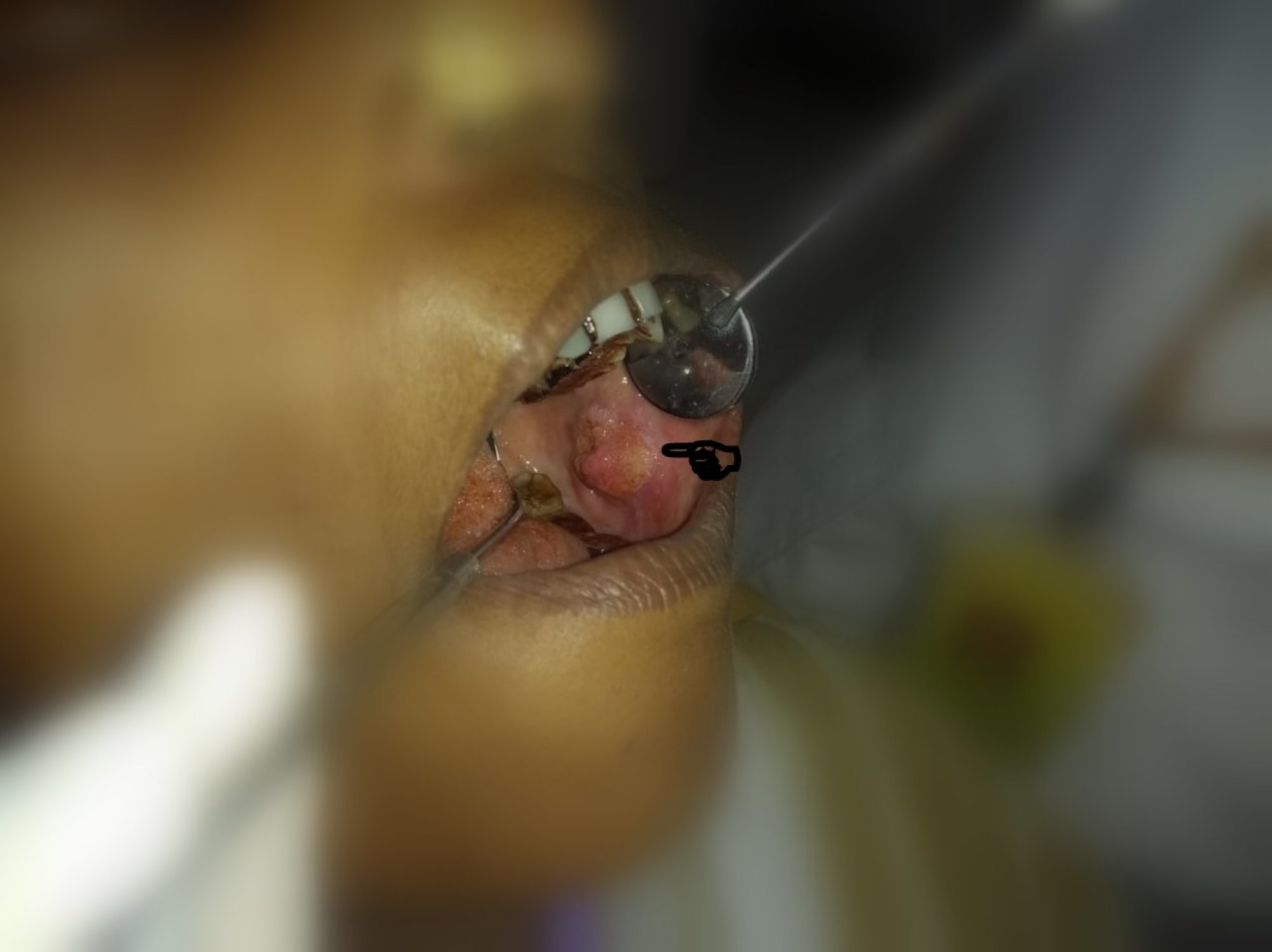
Clinical image of an ulcero-proliferative growth at left mid buccal mucosa region.

A 73-year-old female patient complained of a painful, non-healing growth at her left side inner cheek region for three months. The patient had a history of usage of betel nut since childhood. The patient visited a private dental practitioner one year back for the complaint of sharp tooth at left upper back tooth region and an ulcer at its adjacent mucosal region on the left inner cheek for which she was medicated with a topical anesthetic gel. Intraoral examination revealed a sharp tooth-26, and a 4 x 4 cm tender and indurated ulcero-proliferative growth at left mid buccal mucosa region as shown in Figure 13. Tender, hard and fixed hemispherical-shaped lymph nodes were palpable at the left submandibular region. A provisional diagnosis of malignant non-healing ulcero-proliferative growth was made. Biopsy confirmed well-differentiated SCC. TNM staging: IVA- T4a N1 M0.

## Discussion

All the patients discussed in this presentation were professionally counselled for tobacco cessation and referred to an oncology hospital without further delay, due to their metastasis. Follow-up is done regularly regarding their radiotherapy scheduling and treatment plan.

Sankaranarayanan noted that the male to female ratio of oral cancer in India was 2:1 [6]. Many recent studies showed an increase in the incidence of oral cancer in Indian females, especially those involving the posterior region of oral cavity [7]. In the present report of five cases, two patients were females which can also be correlated with the above prediction.

It has been observed that patients with oral or oropharyngeal malignancy often develop second malignancy or multiple premalignant lesions. This finding led to the theory of field cancerization, which hypothesizes that ‘the entire epithelial surface of the upper aerodigestive tract has an increased risk for development of malignant and premalignant lesions because of multiple genetic abnormalities in the whole tissue exposed to the same carcinogen’ [8]. Hence it is always wise to understand that head and neck malignancy is not a regional mucosal disease, but rather a ‘panmucosal disease’ of the aero-digestive tract. In the present report, one patient had previously done histopathology which suggested features of early malignancy, for which the patient was put on medication by a local village doctor with limited medical facility. This history and the clinical presentation of the patient is in line with the theories of field cancerization, a higher possibility of suspicion.

Several genes are believed to play a key role in carcinogenesis. Tumorigenic effects of some viral infections, especially with the oncogenic HPV subtypes, have also been documented particularly in oropharyngeal cancer [9]. In our present report one patient had a positive first-degree family history for oral cancer along with a history of usage of tobacco, thus a definite dual etiology to suspect; a combination of tobacco and genetics for the initiation of oral malignancy. Patients with no potential risk factors must be further evaluated to find a possible etiology. In the present report, one female patient did not have history of any known potent carcinogens and no family history of any malignancy, hence a possibility to associate the disease with HPV infection [9]. One patient had a sharp tooth in association with a chronic non-healing ulcer. This finding emphasises to the dental professionals to rule out any irritation of a sharp tooth, an ill-fitting denture or a sharp overhanging restoration which can induce a chronic non-healing ulcer in oral mucosa.

Further, one patient was diagnosed at a much initial stage, but was unable to receive an ideal treatment due to deficient health care systems in rural areas of Tamil Nadu. This finding emphasises the role of gaps in the knowledge of dentists, early detection and the consideration of an expert’s opinion.

Better survival rate (for a period of five years) is observed in patients diagnosed at an earlier stage (I and II), compared to later stages. An increased risk of loco-regional failure occurs, if the treatment is delayed by more than a month, in cases of early stage head and neck carcinoma [10].

Our report described five different clinical presentations with an array of etiologies involved and an extensive radiological assessment of histopathologically proven cases of OSCC in rural belt of Tamil Nadu with special emphasis on patient counselling, awareness and early detection to improve quality of life. In this context, this report is an important addition to the existing literature.

## Conclusions

In majority of cases, oral cancer is a preventable disease.Early diagnosis is very important, guiding in the treatment plan, which further improves the survival probability, thus enhancing the quality of life. Hence, dental practitioners in rural set up should be given special training to deal with the varied clinical presentations and radiological forms and to detect oral cancer in their routine practice and refer it to an expert. The duty of a dentist also includes patient counselling, follow-up, creating awareness of ill-effects of tobacco products and in mass screening. Educating the general population about oral cancer is a must to combat mortality and morbidity arising from it. Also, tobacco cessation programme should be implemented by policy makers, social organisations and government missionaries.
